# Real-Time Structural Monitoring of the Multi-Point Hoisting of a Long-Span Converter Station Steel Structure

**DOI:** 10.3390/s21144737

**Published:** 2021-07-11

**Authors:** Yunfeng Zhu, Yi Gao, Qinghe Zeng, Jin Liao, Zhen Liu, Cuiying Zhou

**Affiliations:** 1 China Southern Power Grid Ehv Power Transmission Company, Guangzhou 510275, China; 15915830309@163.com (Y.Z.); gcjzqh@126.com (Q.Z.); 2 School of Civil Engineering, Sun Yat-sen University, No.135 XinGangXiLu, Guangzhou 510275, China; gaoy267@mail2.sysu.edu.cn (Y.G.); liaoj37@mail2.sysu.edu.cn (J.L.); zhoucy@mail.sysu.edu.cn (C.Z.); 3 Guangdong Engineering Research Centre for Major Infrastructure Safety, Guangzhou 510275, China

**Keywords:** long-span, converter station steel structure, hoisting, real-time collaboration, structural monitoring

## Abstract

In the process of using a long-span converter station steel structure, engineering disasters can easily occur. Structural monitoring is an important method to reduce hoisting risk. In previous engineering cases, the structural monitoring of long-span converter station steel structure hoisting is rare. Thus, no relevant hoisting experience can be referenced. Traditional monitoring methods have a small scope of application, making it difficult to coordinate monitoring and construction control. In the monitoring process, many problems arise, such as complicated installation processes, large-scale data processing, and large-scale installation errors. With a real-time structural monitoring system, the mechanical changes in the long-span converter station steel structure during the hoisting process can be monitored in real-time in order to achieve real-time warning of engineering disasters, timely identification of engineering issues, and allow for rapid decision-making, thus avoiding the occurrence of engineering disasters. Based on this concept, automatic monitoring and manual measurement of the mechanical changes in the longest long-span converter station steel structure in the world is carried out, and the monitoring results were compared with the corresponding numerical simulation results in order to develop a real-time structural monitoring system for the whole long-span converter station steel structure’s multi-point lifting process. This approach collects the monitoring data and outputs the deflection, stress, strain, wind force, and temperature of the long-span converter station steel structure in real-time, enabling real-time monitoring to ensure the safety of the lifting process. This research offers a new method and basis for the structural monitoring of the multi-point hoisting of a long-span converter station steel structure.

## 1. Introduction

The longer the span of a converter station steel structure, the more serious the bending deformation, and the higher the risk of fracture or collapse. Globally, many engineering disasters have been caused by the fracturing of converter station steel structures during hoisting [1,2,3]. Deflection, stress, and strain are important mechanical parameters to monitor during the bending failure of long-span structures. Some scholars have performed a considerable amount of research on the structural monitoring of long-span converter station steel structures [4,5]. There have been few long-span converter station steel structure projects worldwide, and, as such, no relevant hoisting experience can be referenced. To avoid the occurrence of disasters in the hoisting process of long-span structures, it is necessary to carry out real-time structural monitoring of the hoisting process.

Research on the structural monitoring of long-span structures has mainly focused on two aspects: analysis control and monitoring methods. In terms of analysis and control, some scholars have developed monitoring systems to process monitoring data in order to analyze and control the construction process of long-span converter station steel structures [6,7]. Xiao et al. [8] established a long-span converter station steel structure health monitoring system that is suitable for cold conditions and treated the temperature parameters of the long-span converter station steel structure as mechanical parameters to guide the construction process. Zhang et al. [9] analyzed the use of optical fiber signals to further monitor long-span structures and proposed a long-span converter station steel structure monitoring system based on an optical fiber signal. However, the temperature and the fiber signal are greatly affected by the environment. To explore more accurate monitoring methods, Hu et al. [10] designed a long-span converter station steel structure axial force monitoring system based on a vibration sensor. According to string vibration theory, the monitoring frequency was converted into a cable force, and indirect measurement of the cable force of a long-span structure was realized at Tongwamen Bridge. Li et al. [11] established a long-span converter station steel structure monitoring system based on a pure circuit and analyzed and controlled the construction of a long-span structure by real-time analysis and processing of electrical signals. Commonly used monitoring methods mainly include automatic monitoring, manual measurement, and comparison with numerical simulation. Sun et al. [12] and Guo et al. [13] studied strain sensor data to monitor long-span converter station steel structures. Li et al. [14] proposed a method to monitor the deflection of a beam, inclination angle of a column, and displacement of a tenon joint in a structure with sensors. Based on the results of nonlinear finite element analysis, Hu and Su [15] derived an estimation formula for the confining pressure in a long-span converter station steel structure. Pagoulatou et al. [16] used the formula proposed by Hu and Su [15] to simulate the mechanical properties of a long-span converter station steel structure. Liang [17] determined the correct formula from the three formulas given by Hu and Su [15] to accurately calculate the confining pressure of a long-span converter station steel structure. Hassanein et al. [18] and Wang et al. [19,20] used the finite element method to predict the compressive performance of a long-span converter station steel structure. Wang et al. [19,20] predicted the bearing capacity of a long-span converter station steel structure by numerical simulation. Other scholars carried out long-term manual measurements for long-span structural engineering. Robertson [21] carried out 9-year manual measurements for the North Halawa Valley Viaduct on the Hawaiian island of Oahu, while Bazant et al. [22] compiled an 18-year field measurement report for the Koror Babeldaob Bridge.

To solve the above problems, the deflection, stress, strain, wind force, and temperature change of the longest long-span converter station steel structure in the world were monitored by automatic monitoring and manual measurement and compared with numerical simulation results. Combined with the monitoring results, a real-time structural monitoring system for the multi-point hoisting of a long-span converter station steel structure is developed, thus ensuring the smooth hoisting process of a long-span converter station steel structure and verifying the reliability of the monitoring system.

## 2. Research Contents and Methods

### 2.1. Project Overview

**Figure 1:**
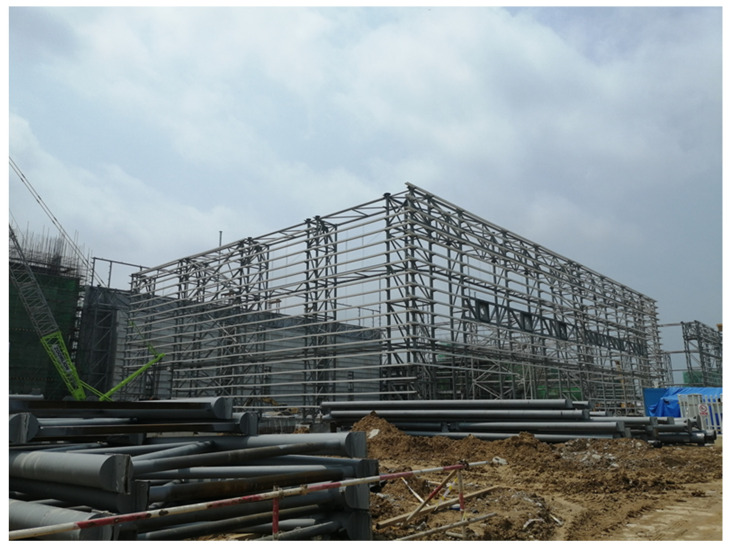
Converter station steel structure site.

The hoisting of a long-span converter station steel structure (including the roof of the long-span converter station steel structure) in South China, which is the longest long-span converter station steel structure in the world, was studied. South China is flanked by the South China Sea, where typhoons occur frequently. Due to its extremely long span, the considered long-span converter station steel structure is more vulnerable to typhoons. Therefore, this long-span converter station steel structure must have a good wind force resistance in order to ensure construction safety. Due to its low latitude and high temperature, the impact of high temperature on the construction of this long-span converter station steel structure was also considered important. The roof adopts a three-layer diagonal pyramid grid structure, while the connections between grid members mostly adopt bolt ball joints. The lower chord of the grid frame has a transfer beam, equipment lifting beam, and crane beam. The transfer beam, equipment lifting beam, and crane beam are set at the bottom chord of the grid structure. The maximum span of the long-span converter station steel structure is 89.5 m, and the weight of the hoisted converter station steel structure is 755 t. A total of 12 lifting points were designed, and multi-point lifting was adopted, as shown in Figure 1.

**Figure 2:**
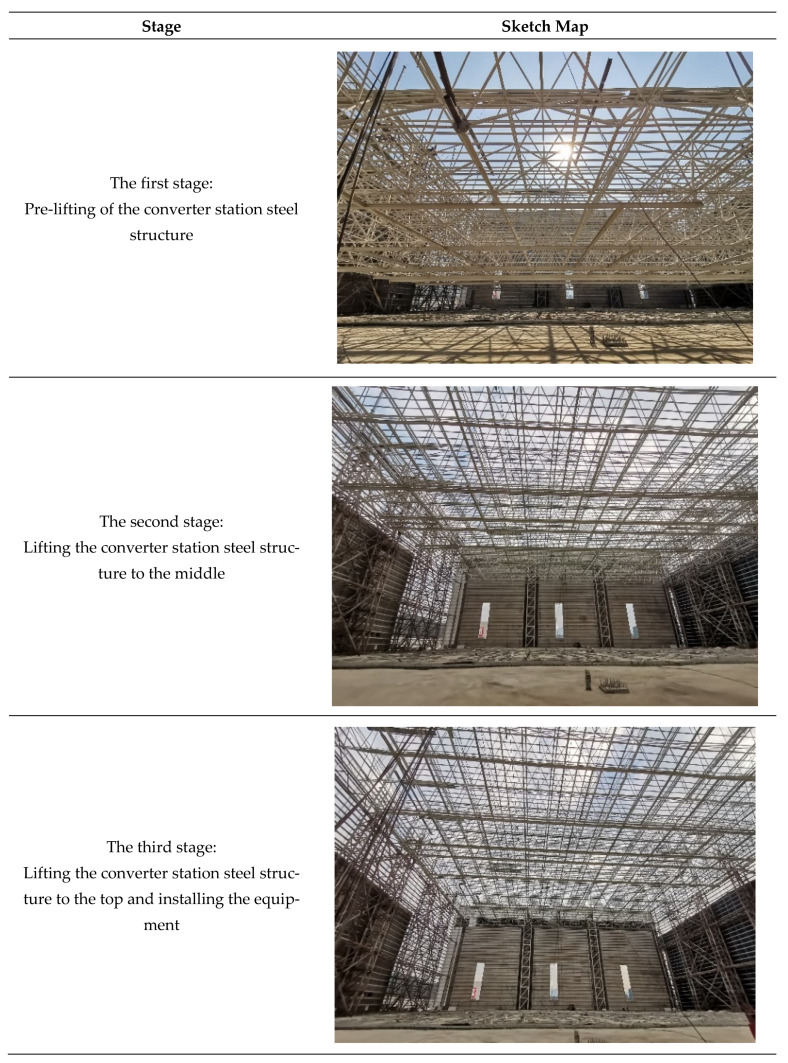
Hoisting stage.

The hoisting of the long-span converter station steel structure is divided into three stages: Pre-lifting of the converter station steel structure, lifting of the converter station steel structure, and lifting of the converter station steel structure to the top, and installation of the 322.7 t of equipment, as shown in Figure 2. In this paper, structural monitoring of the long-span converter station steel structure during the hoisting process is carried out using automatic monitoring and manual measurement, and the results are compared with numerical simulation results.

### 2.2. Multi-Point Hoisting Monitoring Scheme for a Long-Span Converter Station Steel Structure

**Figure 3:**
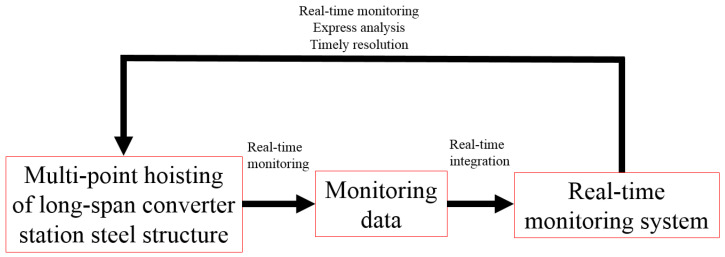
Monitoring principle.

The method of automatic monitoring, manual measurement, and comparison with numerical simulation is used to monitor hoisting in real-time, and the monitoring data are imported into a real-time structural monitoring system. The monitoring system integrates the monitoring data and outputs real-time mechanical two- and three-dimensional images of the converter station steel structure. Through these real-time monitoring images, the construction process is quickly analyzed to help make timely decisions regarding engineering problems that arise, as shown in Figure 3.

**Figure 4:**
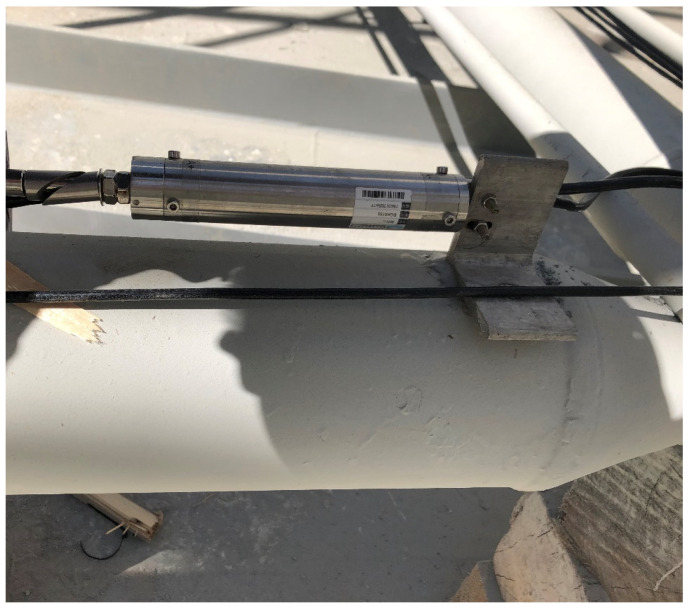
Horizontal single-axis inclinometer.

Automatic monitoring is divided into two parts: deflection monitoring and strain monitoring. Horizontal single-axis inclinometers were mainly used for deflection monitoring, as shown in Figure 4.

**Figure 5:**
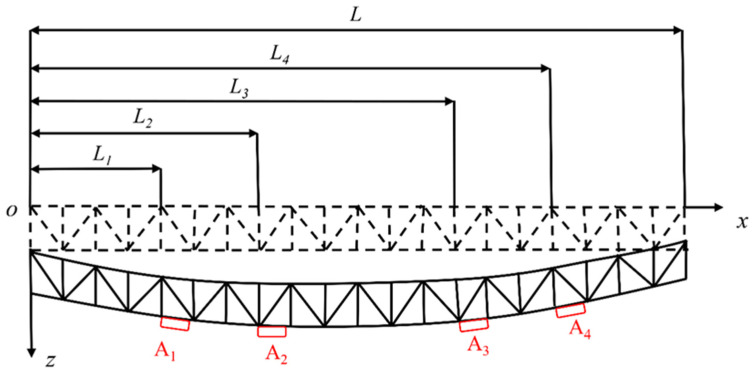
Layout scheme for deflection monitoring.

The layout scheme of each monitoring section (red) is shown in Figure 5.

**Figure 6:**
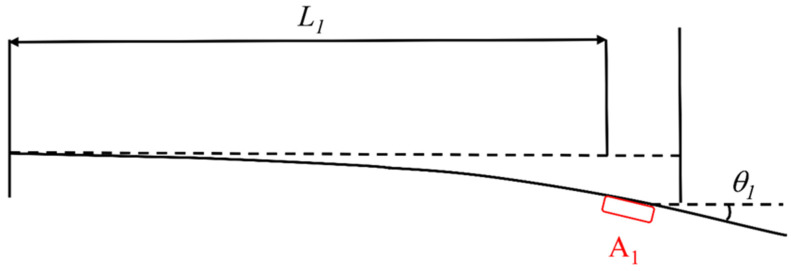
Principle of deflection monitoring.

The monitoring instruments were arranged according to Figure 5 and Figure 6, and the deflection, stress, and strain changes in the hoisting process were monitored, according to the monitoring frequency, once every 10 min. Steel structure hoisting started at 10:00 and ended at 16:00; thus, the whole hoisting process lasted 6 h.

When the inclinometer is stationary, there is no acceleration in the lateral and vertical directions, such that only the acceleration of gravity acts on it. The inclinometer is equipped with two sensors: one for the vertical axis of gravity, and another for the sensitive axis of acceleration. Once the inclinometer produces acceleration, the angle between the vertical axis of gravity and the axis of acceleration sensitivity is taken as the angle of inclination.

Take point *o* as the origin, the vertical direction as the z-axis, and the horizontal direction as the x-axis. The abscissae of the four inclinometers A_1_, A_2_, A_3_, and A_4_ are *L*_1_, *L*_2_, *L*_3_, and *L*_4_, respectively. When the long-span steel structure is forced to bend, the inclinometer will produce acceleration. Through the inclinometer, the bending angle, *θ*, of the long-span converter station steel structure was obtained in order to further obtain the deflection of the long-span converter station steel structure, as shown in Figure 6. The measuring range of the horizontal single-axis inclinometer was ±30°, and the accuracy was 0.01°.

Then, the deflection of the monitoring point is:

(1) $$L_{1}\times \tan\theta_{1}.$$

**Figure 7:**
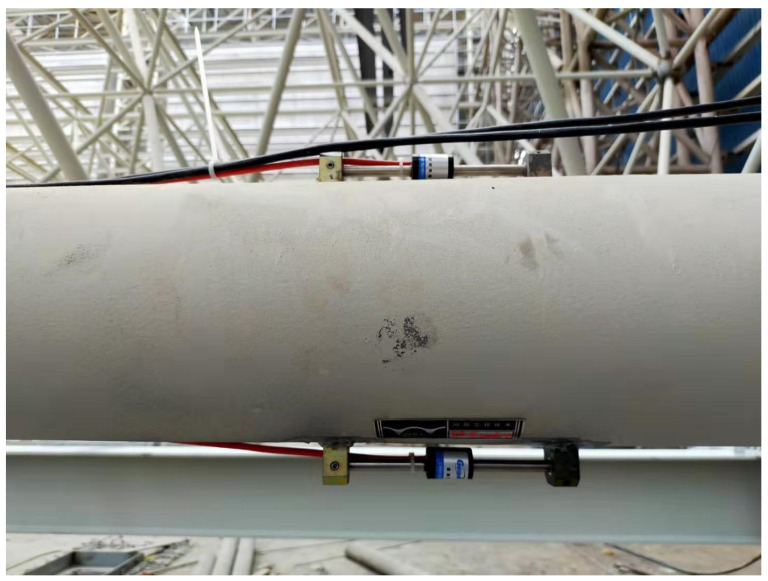
A vibrating wire surface strain gauge.

**Figure 8:**
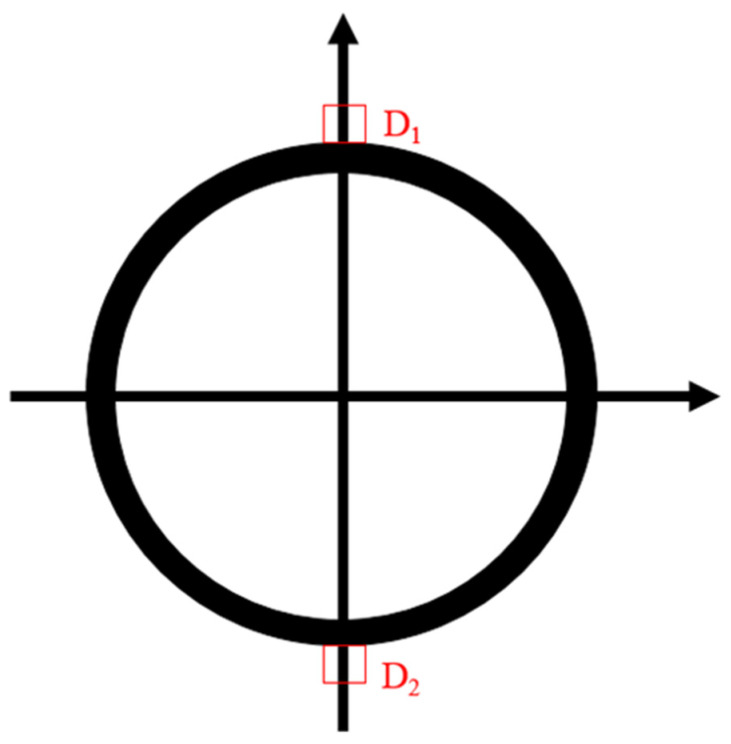
Layout scheme for strain monitoring.

Vibrating wire surface strain gauges were used to measure the strain of the long-span converter station steel structure. Vibrating wire surface strain gauges D1 and D2 were installed on the cross-section of the converter station steel structure in order to monitor the strain changes during the hoisting process of the converter station steel structure. In total, 66 strain gauges were installed on the converter station steel structure. The measuring range of the vibrating wire surface strain gauge was ±1500 με, and the accuracy was 0.1 με, as shown in Figure 7 and Figure 8.

**Figure 9:**
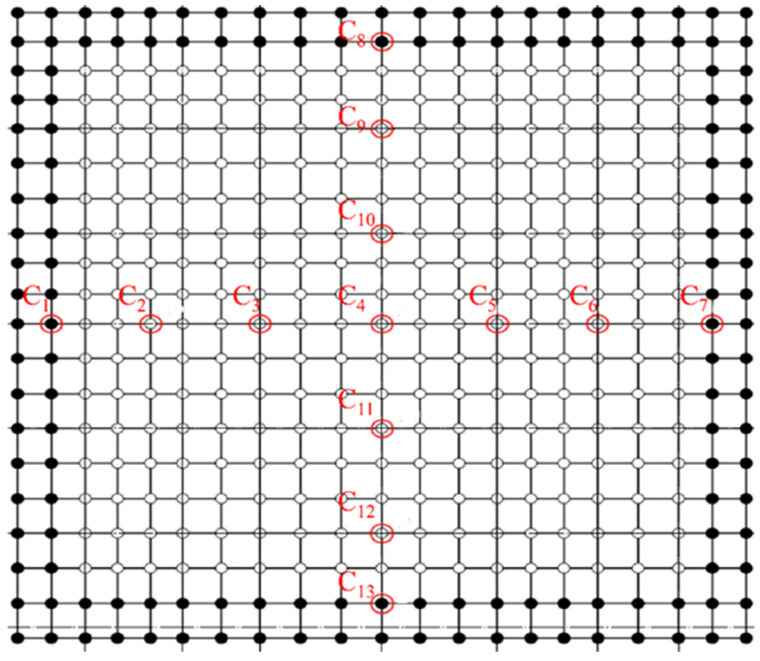
Manual survey control points.

**Figure 10:**
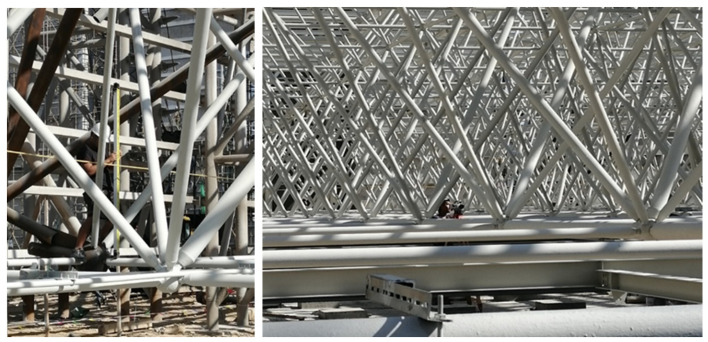
Examples of manual measurement.

The deflection and strain of 13 points, at the same locations as those monitored during the numerical simulation, were measured in the field, as shown in Figure 9. The manual measurement method used a level bar, tower ruler, and level, as shown in Figure 10. The principle of the level bar is that the liquid level is horizontal, while the level bubble is used to directly display the displacement, making it measuring obtain to measure the deviation degree of the relative horizontal, vertical, and inclined position of the measured surface. The length of the level ruler used on site was 250 cm, and the accuracy was 0.02 mm. The tower ruler was used in coordination with the level in order to measure the height difference. The length of the tower ruler used on site was 2 m, and the accuracy was 5 mm. The level was used to provide a horizontal line of sight, with the help of a level ruler with divisions, to directly measure the height difference between two points on the ground, then calculate the unknown point elevation according to the known point elevation and the measured height difference. The accuracy of the level used on site was 1 mm.

In the process of hoisting, manual measurements were carried out every half an hour to measure the mechanical deflection and strain change of the long-span converter station steel structure.

In summary, manual measurements consisted of the use of traditional engineering measuring instruments (e.g., the level ruler, tower ruler, and level) to measure the mechanical parameters of the converter station steel structure as a part of the structural monitoring system. Manual measurement is manual monitoring, while automatic monitoring is fully automatic. Manual measurement captures elevation, which needs to be converted into deflection, while automatic measurement involves the direct output of strain, deflection, and other parameters; furthermore, automatic measurement is more accurate than manual measurement.

### 2.3. Numerical Calculation Model for Multi-Point Hoisting of a Long-Span Converter Station Steel Structure

Unfortunately, the output process of the automatic monitoring results lags, the monitoring operation is complicated, the amount of data is large, and the monitoring efficiency is low. To verify the accuracy of the results and to ensure hoisting safety, the monitoring campaign was supplemented and supported by numerical simulation work, and the hoisting process was further simulated. Considering the complex engineering geological conditions of the project, a finite element model was established to simulate four strata and one converter station steel structure in order to calculate and analyze their stability. The whole model is divided according to the geometry, mesh, boundary constraints, and construction stage.

In the geometric part, the 3D unit entity of four strata was established first; then, the underground pile foundation was driven into the stratum entity according to the construction scheme, and the foundation was engraved on the surface of the entity. Finally, according to the design plan of the large-span converter station steel structure, a 1D line unit of the converter station steel structure is built on the upper part. Through the arrangement of geological survey data, the physical and mechanical parameters of the strata and structural material parameters were set.

In the meshing part, the order of 1D–2D–3D was followed, which is conducive to the generation of nodes from one dimension to three dimensions and prevents the operation from non-convergence. The boundary constraints were divided into two parts: pile constraints and global constraints.

**Figure 11:**
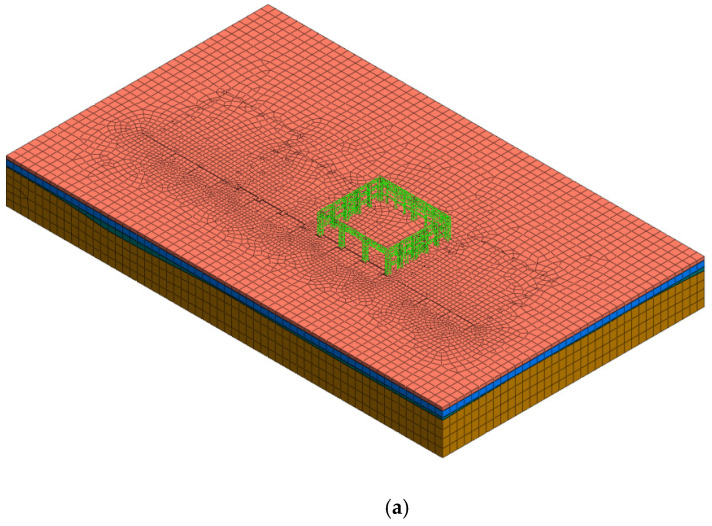

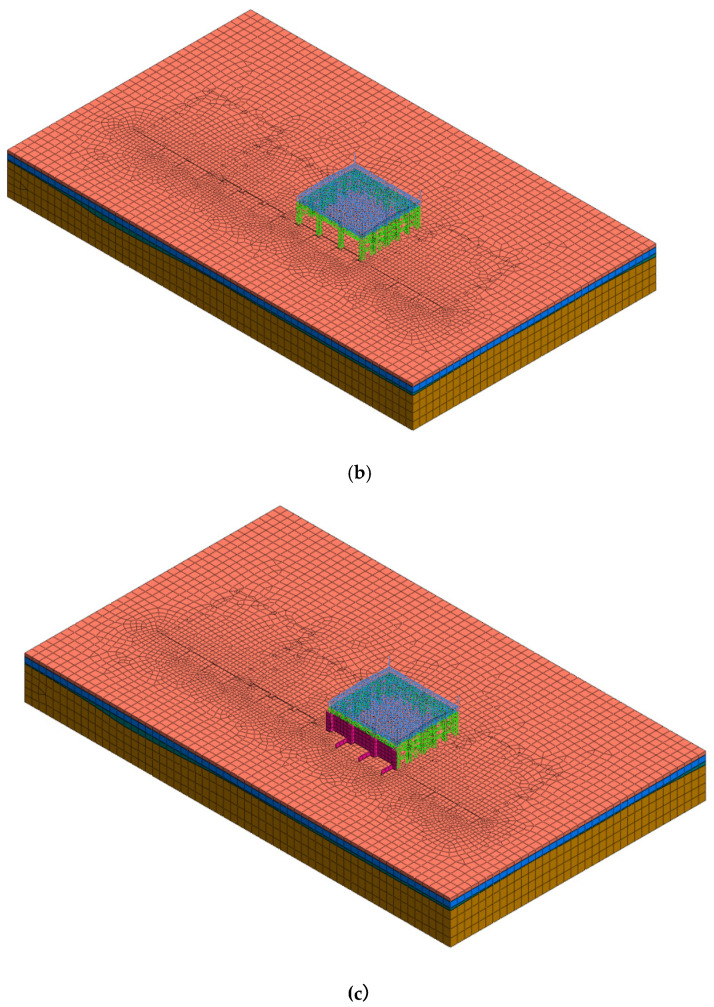
Numerical analysis of converter station steel structure: (**a**) beam and column construction of converter station steel structure, (**b**) hoisting of converter station steel structure, and (**c**) firewall construction.

According to the actual construction sequence, it is divided into an initial stress field, underground pile foundation construction for the converter station steel structure, beam and column construction for the converter station steel structure, first stage hoisting for the converter station steel structure, second stage hoisting of the converter station steel structure, third stage hoisting of the converter station steel structure, and firewall construction. According to the temperature changes over the years in South China, the average minimum temperature was 11 °C, the average maximum temperature was 33 °C, and the annual average temperature was 22 °C. Therefore, to assess the influence of temperature changes on the converter station steel structure, three analysis conditions were proposed: average minimum temperature (10 °C), annual average temperature (22 °C), and mean maximum temperature (33 °C). The boundary constraints were divided into two parts: pile constraints and global constraints. The long-span converter station steel structure was constrained by a semi-rigid connection and semi-hinged connection. The grid size was 2 m. The stability of the converter station steel structure under the three working conditions was analyzed. The model is shown in Figure 11, and the parameters are shown in Table 1, Table 2 and Table 3.

**Figure 12:**
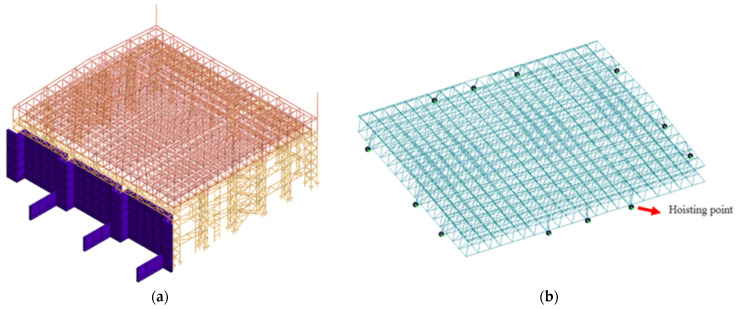
Numerical model: (**a**) converter station steel structure and (**b**) hoisting point.

**Figure 13:**
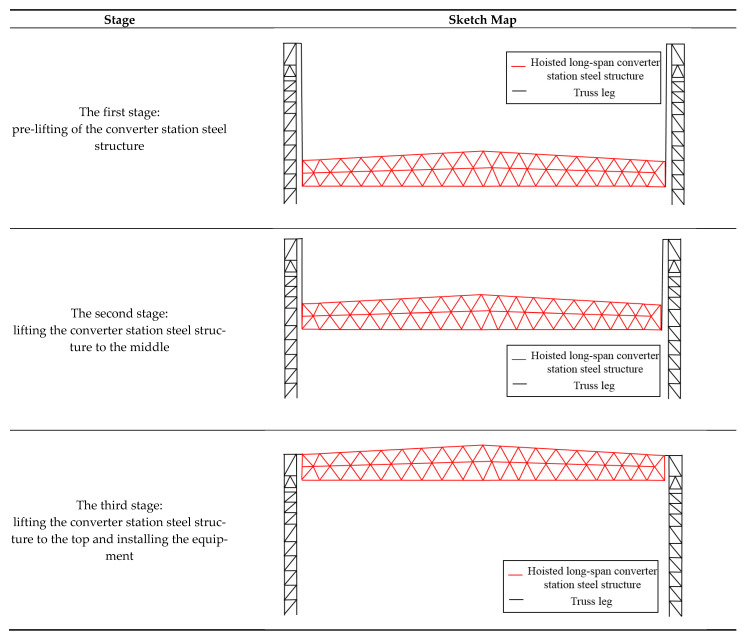
Lifting stage model.

The structural weight of the hoisted converter station steel structure is 755 t, and the model is shown in Figure 12 and Figure 13.

**Figure 14:**
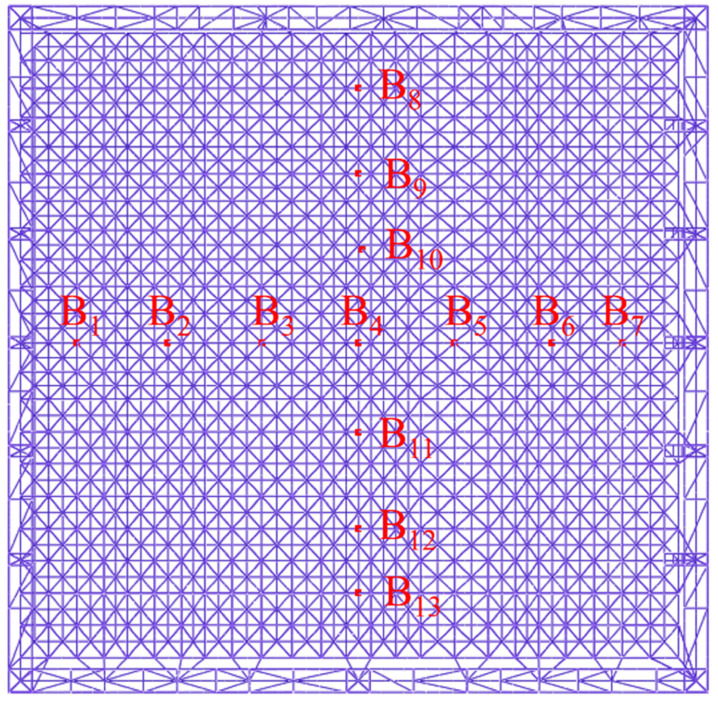
Numerical simulation control point.

Thirteen control points were selected at the bottom of the hoisted steel structure to obtain the deflection and stress. The selected points are shown in Figure 14.

### 2.4. Real-Time Structural Monitoring System for Multi-Point Hoisting of a Long-Span Converter Station Steel Structure

In previous engineering cases, a monitoring system has never been used to monitor the steel structure of a long-span converter station steel structure; thus, no relevant hoisting experience can be referenced. Traditional monitoring methods are used in a small range of applications, and it is difficult to coordinate monitoring and construction control. In the multi-point hoisting of a long-span converter station steel structure, there are many hoisting points, the installation process for the monitoring instruments is complicated, the monitoring results may have errors, and there are potential safety hazards. Monitoring data included the deflection, stress, strain, wind force, and temperature of the long-span converter station steel structure, which change with time. These data were measured every 5 min for 1 year. Routine monitoring needs 9 days to process 1 day’s monitoring data, while the monitoring system only needs 7 min. Through the real-time structural monitoring system, the monitoring and calculation data can be unified for real-time analysis, and the mechanical changes in the long-span converter station steel structure during the hoisting process can be monitored in real-time to achieve the real-time warning of engineering disasters, timely identification of engineering issues, and rapid decision-making, thus avoiding the occurrence of engineering disasters. This real-time structural monitoring system not only can ensure the safety of long-span converter station steel structure hoisting but can also guide subsequent long-span converter station steel structure hoisting projects and provide a reference that is of great significance for the construction of long-span converter station steel structure steel structures.

**Figure 15:**
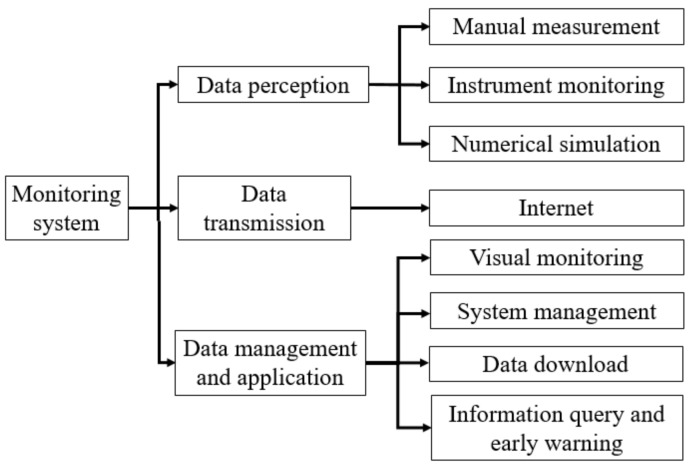
Monitoring system function.

With the help of advanced algorithms, the real-time structural monitoring platform can integrate all kinds of information and data collected from the long-span converter station steel structure monitoring and numerical simulation. Through the comprehensive analysis and evaluation of information, the safety and monitoring data of the whole hoisting process for a long-span converter station steel structure can be visualized in real-time, and the alarm warning information can be issued before the long-span converter station steel structure hoisting risk arises. The architecture of the real-time structural monitoring system includes three functional sub-layers: data perception, data transmission, and data management and application, as shown in Figure 15.

The data sensing module mainly monitors changes in the structural deflection, stress, strain, temperature, and wind force of the long-span converter station steel structure through various intelligent sensors and monitoring instruments, and collects the results of the numerical simulation and manual measurement to realize real-time monitoring of the structural change of the long-span converter station steel structure and assist the on-site inspection personnel in monitoring.

The data transmission module transmits all the kinds of monitoring data from the sensing module to the internet in real-time and realizes the interconnection of information between the site and the monitoring system.

The data management and application module mainly include steel structure safety visual monitoring, system management, data downloading, information querying, and pre-alarm information releasing. Monitoring personnel can learn the safety dynamics of the converter station steel structure at any time and anywhere by utilizing the cloud platform through various clients, quickly querying and downloading the data required for steel structure safety assessment, and obtaining monitoring reports with pictures and text. At the same time, users can also obtain early warning information when the structure first changes abnormally, such that the monitoring personnel can take appropriate measures in a timely manner in order to prevent the occurrence of safety accidents. In addition, the monitoring personnel can interact with the patrol personnel in real-time, according to the needs of the monitoring project and the expectations of various emergencies.

After the installation of the monitoring instruments on the long-span converter station steel structure, the construction stage started. On the main page of the monitoring system, three monitoring approaches can be selected—numerical simulation, manual measurement, and automatic monitoring, to view the monitoring data.

**Figure 16:**
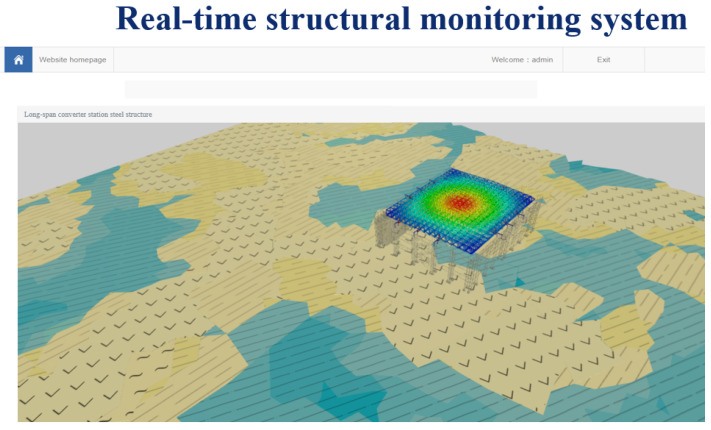
Monitoring system function page.

As shown in Figure 16, after entering the selected module, the stress, strain, deflection, wind force, and temperature change of the long-span converter station steel structure during the lifting process, information can be displayed in real-time, and the visual monitoring, system management, data downloading, information querying and pre-alarm information releasing of the lifting process can be carried out in real-time.

## 3. Results and Discussion

### 3.1. Monitoring Results for the Multi-Point Hoisting of a Long-Span Converter Station Steel Structure

#### 3.1.1. Monitoring Results

**Figure 17:**
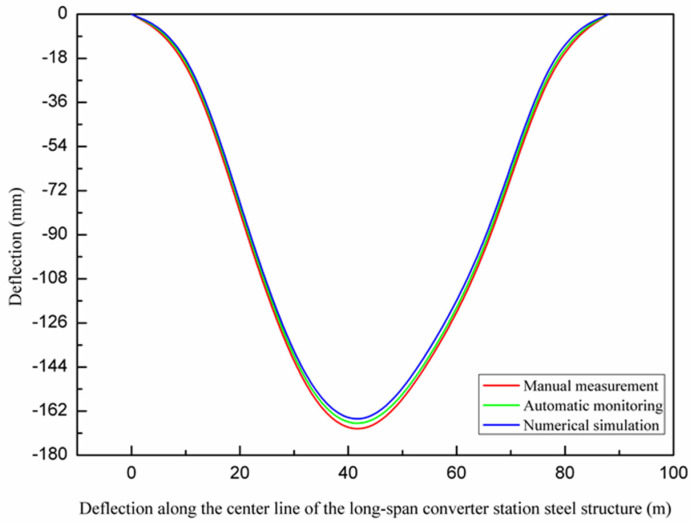
Deflection along the center line of the long−span converter station steel structure.

Through the real-time structural monitoring system, the results of automatic monitoring, manual measurement, and numerical simulation were compared. The maximum deflection values identified from the automatic monitoring, manual measurement, and numerical simulation were all located in the center of the long-span converter station steel structure and gradually decreased towards both ends, as shown in Figure 17.

**Figure 18:**
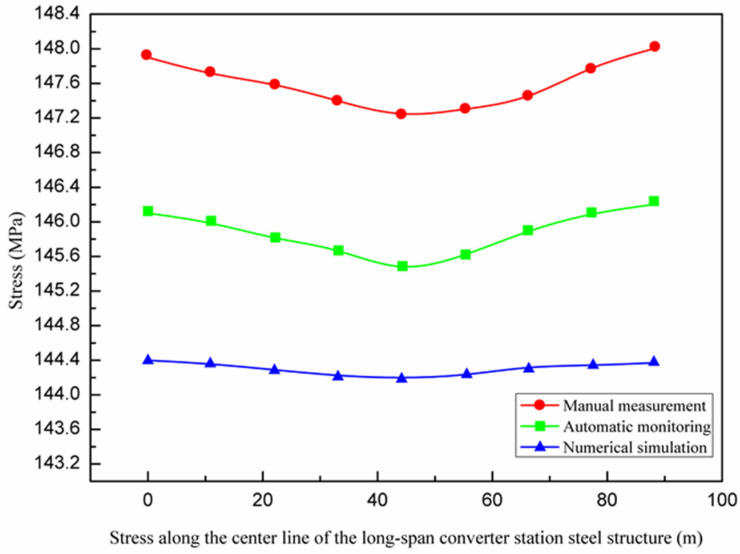
Stress along the center line of the long−span converter station steel structure.

The maximum stress values identified from the numerical simulation, manual measurement, and automatic monitoring were all located at both ends of the converter station steel structure and gradually decrease towards the center, while the overall stress changed little, as shown in Figure 18.

However, as numerical simulation is the result of theoretical calculation, errors can arise, partly as the factors of the field environment are not considered. Manual measurement cannot be synchronized with the hoisting process in real-time, as it will delay the progress of the project and waste time. Therefore, through the real-time structural monitoring system, monitoring, calculation, decision-making, and analysis are integrated, such that the real-time structural monitoring can be used during the whole hoisting process in order to solve the problems associated with the many hoisting points, complex monitoring, large amount of data, low efficiency, and difficulty in management and control during the monitoring process.

#### 3.1.2. Monitoring System Results

**Figure 19:**
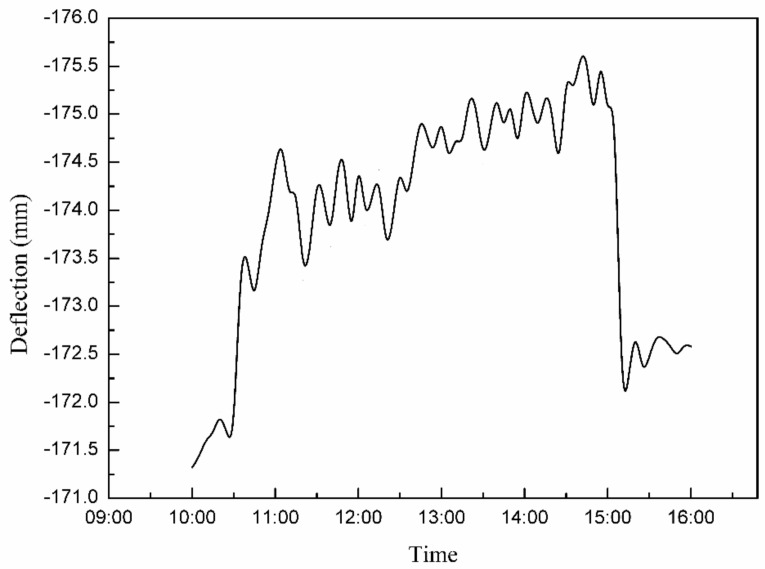
Change in the deflection of the long−span converter station steel structure.

**Figure 20:**
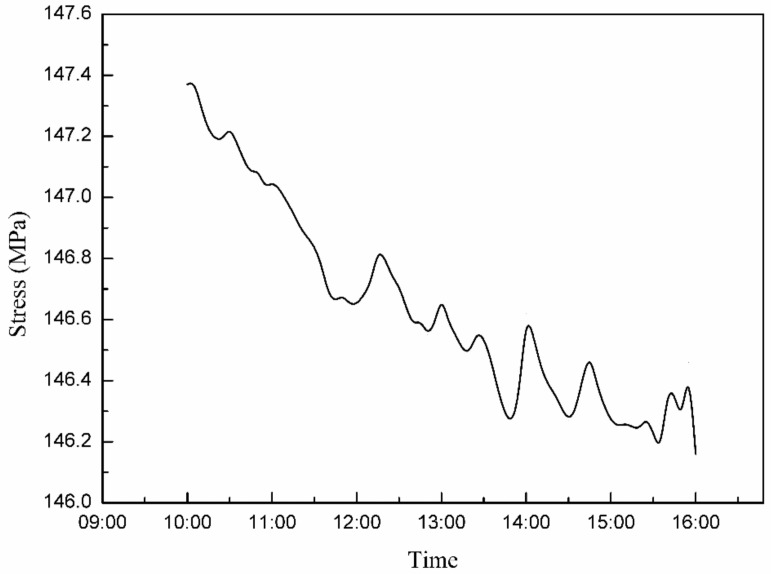
Change in the stress of the long−span converter station steel structure.

Through the monitoring system, the changes in stress, strain, deflection, temperature, and wind force at the monitoring points could be output to obtain the general rule of hoisting and the monitoring images of deflection, stress, strain, temperature, and wind force at certain points, as shown in Figure 19 and Figure 20.

With the monitoring system, the deflection and stress changes of the long-span converter station steel structure can be monitored in real-time during the lifting process, such that on-site lifting and monitoring can be synchronized. When the deflection and stress of the long-span converter station steel structure are close to the critical value of risk, the construction should be stopped in a timely manner for safety investigation, resulting in the real-time warning and the timely treatment of potential safety hazards. During the whole hoisting process, the structural stress and deflection of the long-span converter station steel structure should change smoothly in order to meet the safety specifications.

**Figure 21:**
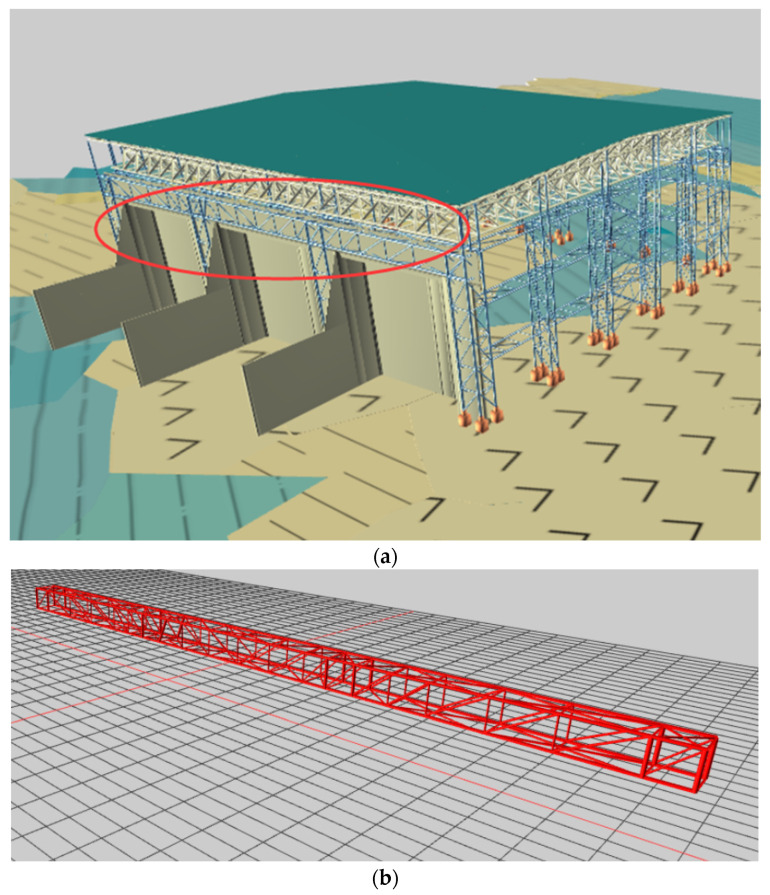
Selection of structural members for the long−span converter station steel structure.

The real-time structural monitoring platform encodes the long-span converter station steel structure, such that the long-span converter station steel structure can be selected in the monitoring platform to immediately visualize its stress and deflection change state. The structural members of the long-span converter station steel structure can be selected separately, as shown in Figure 21 (see red area).

**Figure 22:**
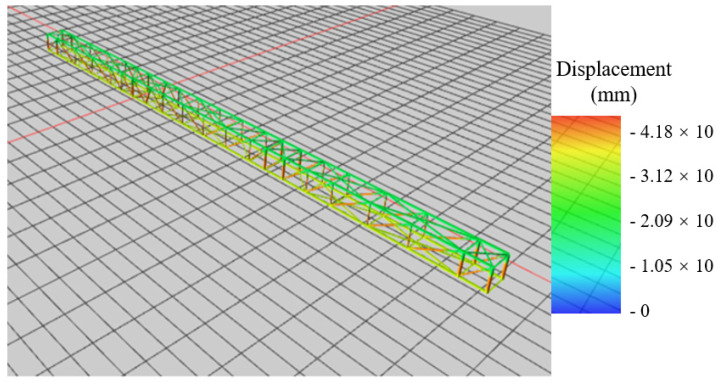
Deflection of selected members of the long−span converter station steel structure.

Any node can be selected to check the stress and deflection of the long-span converter station steel structure, as shown in Figure 22.

**Figure 23:**
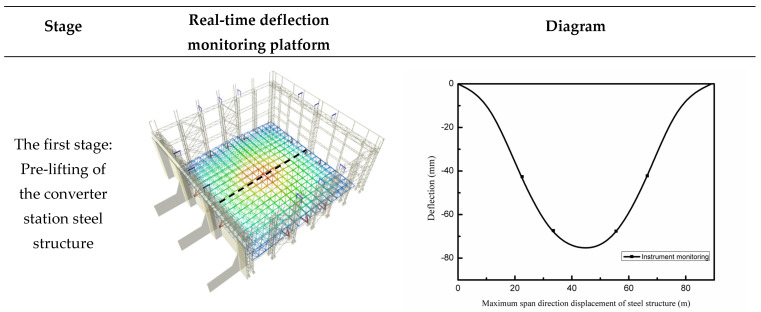

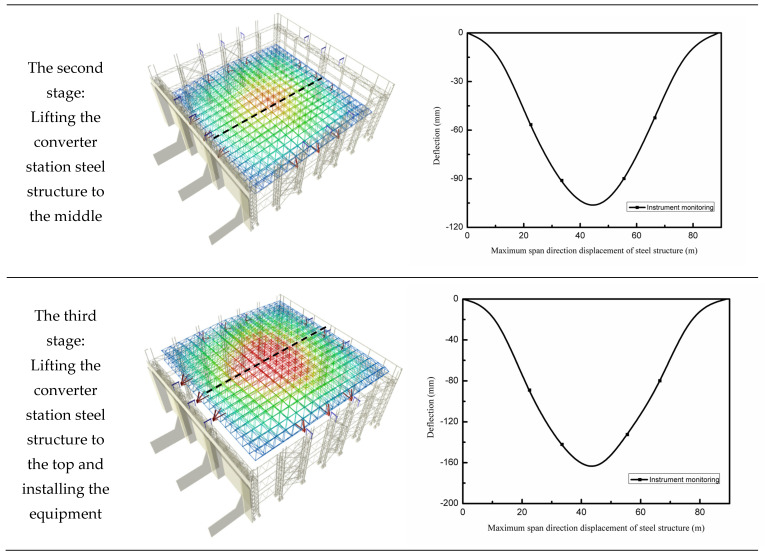
Three−dimensional, real−time monitoring.

Using the characteristics of three-dimensional model visualization, the mechanical changes during project construction are clearly and intuitively displayed, and the three-dimensional lifting image is automatically generated—corresponding to the real-time lifting on site—to monitor the lifting process in real-time and ensure that the construction can be carried out in an organized way according to the design scheme. The real-time deflection monitoring platform is divided into three stages, where the numerical model of each stage corresponds to that of the monitoring system. as shown in Figure 23.

This monitoring system can simulate the maximum deformation of a long-span converter station steel structure in real-time under 17 levels of the wind scale during the hoisting process, which can assist in engineering decision-making, as shown in Table 4.

**Figure 24:**
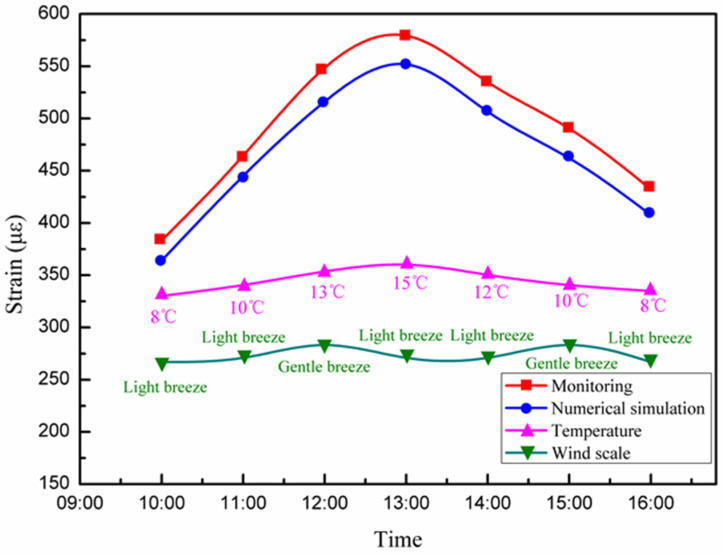
The changes in temperature, wind force, and strain of the long-span converter station steel structure.

The maximum allowable deformation of the studied long-span converter station steel structure is 180 mm, indicating that it can bear the influence of super typhoons (Level 17) in South China. During the construction of the long-span converter station steel structure (i.e., winter in South China), the wind scale was 2–3, which had little influence on the long-span structure. Considering the air temperature in the range of 5–15 °C, the changes in the strain, temperature, and wind force of the long-span converter station steel structure were output by the monitoring system in real-time, as shown in Figure 24.

The strain of the long-span converter station steel structure was 364.1–597.7 με, the wind force was 2–3, and the temperature was 8–15 °C. The strain decreased with increasing temperature, in agreement with the principles of thermal expansion and contraction. The maximum strain of the long-span converter station steel structure occurred at 15 °C, at which time the wind was a light breeze.

### 3.2. Analysis of Multi-Point Hoisting of a Long-Span Converter Station Steel Structure

**Figure 25:**
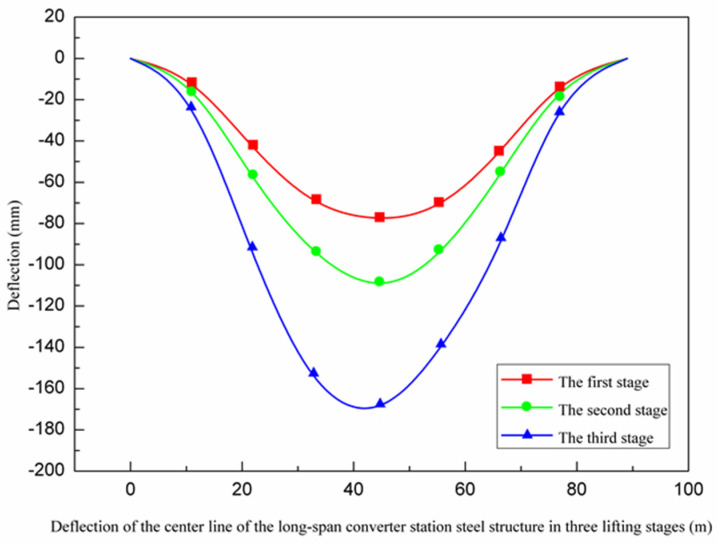
Deflection of the centerline of the long−span converter station steel structure in three lifting stages.

Deflection is an important parameter, which is used to reflect the bending deformation of long-span structures, and which is important to consider when evaluating engineering safety. As shown in Figure 19, from the first stage to the third stage (10:00–15:00), the deflection of the long-span converter station steel structure gradually increased from 81 mm to 86 mm. When the long-span converter station steel structure was placed on the truss of the converter station steel structure at 15:00, the deflection decreased rapidly to 82 mm, and the bending degree of the long-span converter station steel structure first increased slowly, then decreased quickly and ultimately tended to stability. As shown in Figure 17 and Figure 25, the maximum deflection of the three hoisting stages was located at the centerline of the long-span converter station steel structure; the deflections from the first stage to the third stage were 79.2 mm, 114.5 mm, and 175.8 mm, respectively, and the maximum deflection was observed in the third stage. During the construction process, members were installed in the structure in the second and third stages, such that the third stage corresponded to the largest structural load. Therefore, in the multi-point hoisting process of this long-span converter station steel structure, the deflection of the centerline of the long-span converter station steel structure under the maximum load state was the largest, making it the location most prone to engineering disasters. With the structural monitoring system, the centerline of the long-span converter station steel structure under the maximum load state was monitored. Therefore, in future long-span converter station steel structure hoisting projects, it is necessary to focus on monitoring the centerline of the long-span converter station steel structure under the maximum load state in order to ensure safe operations. During the hoisting process, the method of increasing the reverse direction force on the long-span converter station steel structure to offset the load can be used to reduce the deflection of the long-span converter station steel structure and reduce the engineering risk.

**Figure 26:**
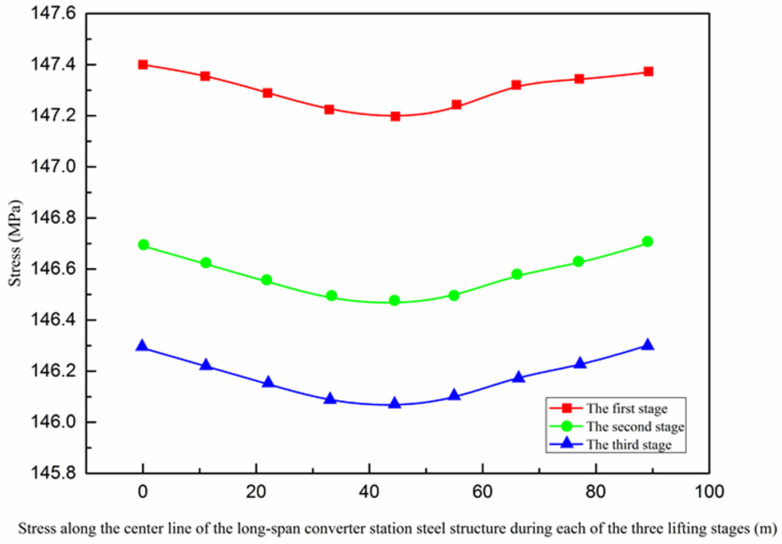
Stress along the centerline of the long−span converter station steel structure during each of the three lifting stages.

The stress of a long-span structure is an important parameter, which reflects the overall stress state and can be used to determine the bearing capacity. From the first stage to the third stage of hoisting, the stress of the long-span structure gradually decreased from 147.4 MPa to 145.06 MPa. According to Figure 18 and Figure 26, the maximum stress values of the three lifting stages were all located near the lifting points on both sides in the long-span converter station steel structure. The maximum stress values of the first to third lifting stages of the long-span converter station steel structure were 147.4 MPa, 146.7 MPa, and 146.3 MPa, respectively, where that of the first stage was the largest. For multi-point hoisting, the area that needs to be focused on during stress monitoring is the hoisting point of the long-span converter station steel structure under the maximum load. In subsequent multi-point hoisting construction processes for the long-span converter station steel structure, the design of the hoisting point scheme and stress monitoring of the hoisting point are the most important design aspects.

**Figure 27:**
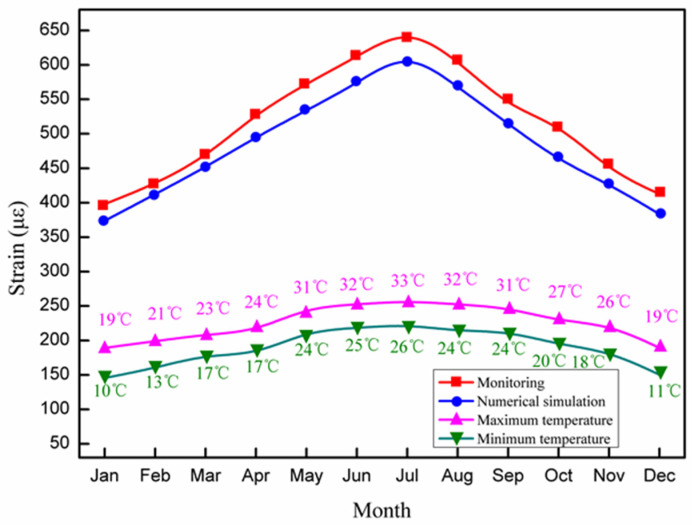
Annual variation in the temperature and strain of the long-span converter station steel structure.

The mechanical properties, wind force, and temperature of the long-span converter station steel structure are important factors affecting the hoisting safety of the considered long-span converter station steel structure in South China, as shown in Figure 27. During the 1-year monitoring period, the structural strain of the long-span converter station steel structure increased with increasing temperature. The maximum value appeared in July, when the temperature was 26–33 °C, while the minimum value appeared in January, when the temperature was 10–19 °C.

The hoisting of traditional short- and medium-span converter station steel structures faces many difficulties. As the spans of short- and medium-span converter station steel structures are less than 30 m, it is inconvenient to install monitoring instruments due to the construction cost. Such converter station steel structures are simple and easily disturbed by external wind, gravity, and other conditions, resulting in monitoring errors. Therefore, the numerical simulation of short- and medium-span converter station steel structures is generally carried out; however, the numerical simulation is the result of only theoretical research, such that the accuracy of the numerical simulation needs to be further tested. This problem can be solved by the use of a real-time structural monitoring system. Through the composite monitoring of short- and medium-span converter station steel structures, real-time monitoring can be realized.

**Figure 28:**
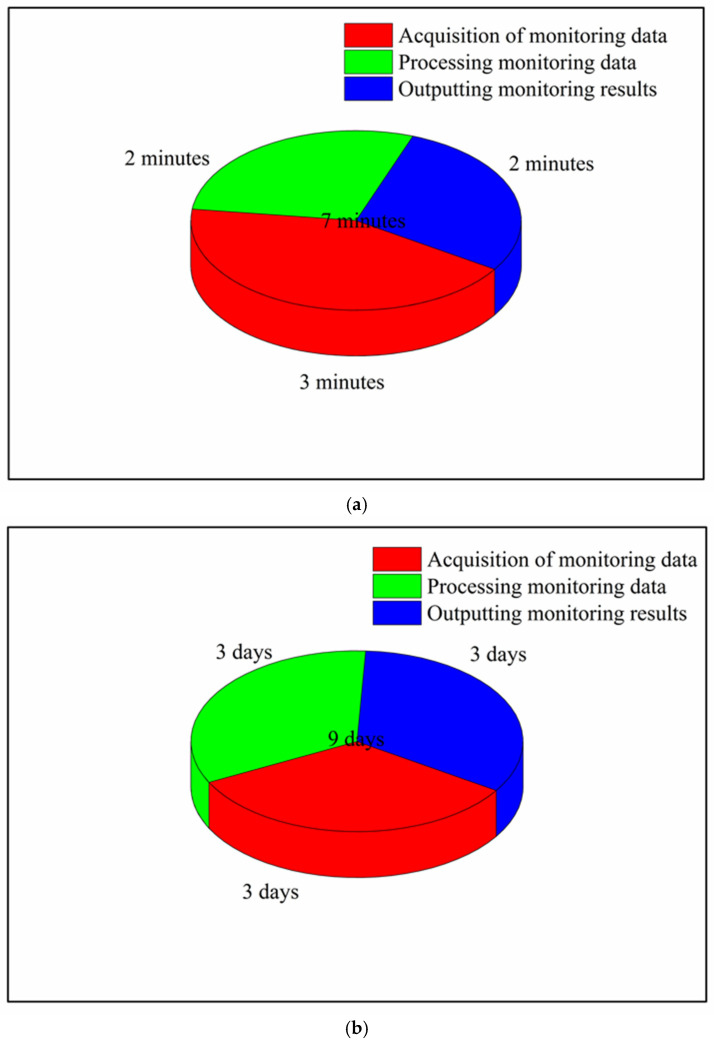
Time consumption of a monitoring cycle: (**a**) proposed real−time structural monitoring system and (**b**) traditional monitoring methods.

Multi-point hoisting of long-span converter station steel structures has rarely been undertaken, such that little reference information can be gleaned from experience. Traditional long-span converter station steel structure automatic monitoring requires a 9-day work cycle from installation to monitoring to final data processing. Compared with the project progress, this results in serious delays and wastes considerable time; however, the proposed real-time structural monitoring system takes only 7 min to output monitoring results. Traditional monitoring methods require costly manual processing and output of the monitoring data, as shown in Table 5 and Figure 28.

Constrained by the limitations of monitoring methods, traditional monitoring also results in certain errors. Through the real-time structural monitoring system, a large amount of data can be processed synchronously in real-time, the desired results can be obtained immediately, a considerable amount of time can be saved, and the cost can be significantly reduced. With the use of computer intelligence, the operation of the monitoring system is simple, and the real-time warning of potential safety hazards can be issued, such that engineering disasters can be identified in a timely manner, decision-making can be carried out quickly, and the occurrence of engineering disasters can be avoided.

The proposed real-time structural monitoring platform can solve the problems related to the small scope of application of traditional monitoring methods and the difficulty in management and control of monitoring and construction. This real-time structural monitoring system unifies the calculation data for real-time analysis. It can process a large amount of monitoring data simultaneously, thus reducing the monitoring time and monitoring error. It can monitor the mechanical changes of long-span converter station steel structures in real-time during the hoisting process in order to achieve real-time warning of engineering disasters, timely identification of engineering issues, and rapid decision making, thus avoiding the occurrence of engineering disasters.

Therefore, compared with traditional monitoring methods, the real-time structural monitoring system can save time and money, as well as greatly reduce the engineering risk. It is necessary to apply such a monitoring system for long-span converter station steel structure hoisting. As the frontier research considering a multi-point hoisting monitoring system for a long-span converter station steel structure, this work can provide not only a reference for the planning of multi-point hoisting for other long-span converter station steel structures but also technical guidance and support for the existing monitoring mode of short- and medium-span converter station steel structure hoisting. Thus, the presented research results are applicable to all converter station steel structures.

## 4. Conclusions

- Based on the hoisting process of a long-span converter station steel structure in South China, this work monitored the structural mechanics of the long-span converter station steel structure by means of automatic monitoring, manual measurement, and comparison with numerical simulation. We established a real-time monitoring system that combines these three monitoring methods. The real-time monitoring data are transmitted to the monitoring system, and monitoring, calculation, analysis, and decision making are integrated in order to synchronously generate visualizations of the changes in deflection, stress, strain, temperature, and wind in real-time, as well as a three-dimensional visualization of the long-span converter station steel structure to truly reflect the on-site lifting situation. When the mechanical properties of the long-span converter station steel structure change suddenly, it can be detected quickly, allowing for real-time warning and response. This approach overcomes the problems of complex operations, large amounts of data, low efficiency, and difficulty of management and control in the traditional long-span converter station steel structure hoisting monitoring method.
- The real-time monitoring system proposed in this paper can monitor not only the deflection, stress, strain, temperature, and wind force of a long-span converter station steel structure but also the bending moment, load, and other parameters, by changing the monitoring instruments.
- The real-time monitoring system proposed in this paper can monitor the hoisting process of a long-span converter station steel structure, and can be used to monitor the construction of public and industrial buildings, such as long-span gymnasiums, cinemas, exhibition halls, city halls, airports, train stations, and wharves, which will provide further experience and guidance for the construction of other long-span structures.

## Figures and Tables

**Table 1:** Parameter table of formation physical and mechanical properties.

Geotechnical Stratification	Geotechnical Name	Compression Modulus (kPa)	Poisson’s Ratio	Bulk Density (kN/m^3^)	Cohes. (kPa)	Friction Angle (°)
<1>	Plain fill	8.0	0.3	18	11	12
<2>	Silty clay	6.89	0.3	18.6	20.9	19.3
<3>	Strongly weathered silty mudstone	10.0	0.358	21	32.5	26.3
<4>	Moderately weathered silty mudstone	12.0	0.337	21	35	28

**Table 2:** Table of structural material parameters.

Material Name	Modulus of Elasticity (GPa)	Poisson’s Ratio	Cohes. (kPa)
Concrete	28	0.2	24
Pile foundation	28	0.2	24
Upper steel structure	206	0.28	78

**Table 3:** Comparison table of wind level, wind speed, and wind load.

Wind Scale	Name	Wind Speed (m/s)	Maximum Wind Pressure (kPa)	Wind Load (kPa)
5	Fresh breeze	8.0–10.7	0.000716	0.19
7	Gale	10.8–13.8	0.00119	0.49
9	Gale	13.9–17.1	0.001828	1.0
11	Storm	17.2–20.7	0.002678	1.79
12	Hurricane	20.8–24.4	0.003721	2.29
13	Typhoon	24.5–28.4	0.005041	2.89
14	Violent typhoon	28.5–32.6	0.006642	3.58
15		32.7–36.9	0.00851	4.36
16	Super typhoon	37.0–41.4	1.00072	5.28
17		41.5–46.1	1.00328	6.31

**Table 4:** The maximum deformation of the long-span converter station steel structure in the process of hoisting, considering 17 levels of wind scale.

Wind Scale	Name	Wind Load (kPa)	Maximum Deformation of Long-Span Converter Station Steel Structure (mm)
5	Fresh breeze	0.19	2.35
7	Gale	0.49	6.055
9	Gale	1.0	12.36
11	Storm	1.79	22.12
12	Hurricane	2.29	28.30
13	Typhoon	2.89	35.71
14	Violent typhoon	3.58	44.24
15		4.36	53.88
16	Super typhoon	5.28	65.24
17		6.31	77.97

**Table 5:** Comparison between the control system and the traditional monitoring system.

Evaluation Parameters	Monitoring System	Routine Monitoring
Monitoring time	7 min	9 days
Data-processing method	Real-time mass processing	Manual treatment
Early warning mechanism	Efficient and fast	None
Operation mode	Intelligent operation	Slow and inefficient
Analysis results	Real-time synchronous output	Manual output

## Data Availability

Data is contained within the article.
